# Grenzbereiche der Thrombektomie

**DOI:** 10.1007/s00115-021-01138-5

**Published:** 2021-06-07

**Authors:** Marios-Nikos Psychogios, Alex Brehm, Peter Sporns, Leo H. Bonati

**Affiliations:** 1 Abteilung für interventionelle und diagnostische Neuroradiologie, Klinik für Radiologie und Nuklearmedizin, Universitätsspital Basel, Petersgraben 4, 4031 Basel, Schweiz; 2 Hirnschlagzentrum, Klinik für Neurologie, Universitätsspital Basel, 4031 Basel, Schweiz

**Keywords:** Ischämischer Schlaganfall, Mechanische Thrombektomie, Endovaskuläre Therapie, Gefäßverschluss, Behandlungsindikationen, Ischemic stroke, Mechanical thrombectomy, Endovascular treatment, Vascular occlusion, Treatment indications

## Abstract

Die mechanische Thrombektomie (MT) hat sich als Standardverfahren für die Behandlung akuter ischämischer Schlaganfälle aufgrund eines Verschlusses einer großen, proximalen Hirnarterie der vorderen Zirkulation etabliert. Dennoch sind nach aktuellen Guidelines noch große Patientenkollektive von dieser hocheffektiven Behandlungsmethode ausgeschlossen. Diese Arbeit gibt daher einen Überblick über mögliche Erweiterungen der Behandlungsindikationen für die MT, wie z. B. Patienten im erweiterten Zeitfenster, mit distalen Verschlüssen, mit großem Infarktkern oder auch für sehr alte (> 90 Jahre) und junge (0–17 Jahre) Patienten. Zusätzlich besprechen wir neue Entwicklungen in der interventionellen Behandlung von Schlaganfällen, wie z. B. neue Triage-Konzepte oder die Fragestellung, ob die zusätzliche intravenöse Thrombolyse bei MT-Patienten notwendig ist. Abschließend geben wir für die besprochenen Behandlungsindikationen unsere Einschätzungen basierend auf der aktuellen Literatur und unserer klinischen Erfahrung.

## Abstract (en)

Mechanical thrombectomy (MT) has become the standard procedure in the treatment of patients with acute ischemic stroke (AIS) due to occlusion of a large proximal cerebral artery of the anterior circulation. Nevertheless, according to the current guidelines large patient collectives are still excluded from this highly effective treatment method. Therefore, this article gives an overview of possible extensions of the indications for treatment with MT. For example, patients in the extended time window with distal occlusions, with large infarct cores and also for very old (90+ years) or young (0–17 years) patients. Furthermore, we discuss recent developments in the interventional treatment of stroke, such as new triage concepts or the question whether an additional intravenous thrombolysis is necessary in patients with MT. We conclude with our own estimations for the discussed indications for treatment based on our clinical experience and the current literature.

## Hintergrund

Die mechanische Thrombektomie (MT) wurde im Jahr 2015 nach der Veröffentlichung von fünf unabhängigen randomisiert-kontrollierten Studien (RCTs) das Standardverfahren für die Behandlung eines akuten ischämischen Schlaganfalls (AIS) aufgrund eines proximalen Arterienverschlusses („large vessel occlusion“, LVO) der vorderen Zirkulation im Zeitfenster von 0–6 h. Der klinische Nutzen war mit einer „number needed to treat“ von ca.  2,5 für die Reduktion neurologischer Langzeitfolgen überwältigend [1]. Allerdings führten die strengen Einschlusskriterien der oben genannten Studien dazu, dass nur ca. einer von 17 Schlaganfallpatienten sich für diese Behandlung qualifizierte [2]. Aktuelle Studien weisen darauf hin, dass der Anteil an Schlaganfallpatienten, die von der MT profitieren könnten, mit ca. 22 % deutlich höher ist [3, 4].

In diesem Review geben wir daher einen Überblick über die aktuelle Literatur insbesondere im Hinblick auf Patientenkollektive, die in den bisherigen RCTs unterrepräsentiert waren. Zusätzlich beleuchten wir aktuelle Neuentwicklungen in der interventionellen Behandlung von AIS-Patienten. Abschließend fassen wir die Empfehlungen der aktuellen Guidelines der amerikanischen Herz- und Schlaganfallorganisation (American Heart Association/American Stroke Association, AHA/ASA) und der europäischen Schlaganfallorganisation (ESO) im Hinblick auf die oben angesprochenen Patientenkollektive zusammen [5, 6]. Ergänzend geben wir auch eine eigene Empfehlung auf Basis der hier besprochenen Literatur (Tab. 1).

**Table 1:** 

Indikation	ESO/ESMINT-Guidelines [6]	AHA/ASA-Guidelines [5]	Aktuelle Forschungsergebnisse und Empfehlung der Autoren
Zeit von Symptombeginn/zuletzt gesund gesehen zum Behandlungsbeginn	Bei Patienten mit einem Verschluss der vorderen Zirkulation, die sich zwischen 6 und 24 h, nachdem sie zuletzt gesund gesehen wurden, präsentieren, wird eine MT empfohlen, sofern die DAWN- oder DEFUSE-3-Kriterien erfüllt sind	Bei Patienten mit AIS aufgrund eines Verschlusses der vorderen Zirkulation, die sich zwischen 6 und 16 h nach Symptombeginn, nach dem sie zuletzt gesund gesehen wurden, vorstellen und die DAWN- oder DEFUSE-3-Kriterien erfüllen wird eine Thrombektomie empfohlen	Auch in einem Zeitfenster von > 24 h sollte eine Thrombektomie erwogen werden, sofern in der (Perfusions‑)Bildgebung noch eine relevante Penumbra nachgewiesen werden kann
	*Grad der Evidenz: moderat ⊕⊕⊕*	*Grad der Evidenz: A*	
	*Stärke der Empfehlung: stark ↑↑*	*Stärke der Empfehlung: stark ↑↑*	
	Expertenmeinung: Im Zeitfenster von 6–12 h können Patienten, die die ESCAPE-Kriterien (moderate bis gute Kollateralen und ASPECTS ≥ 6) erfüllen durch MT behandelt werden	Bis zu 24 h ist eine mechanische Thrombektomie bei Patienten, die die DAWN Kriterien erfüllen angemessen	
		*Grad der Evidenz: B‑R*	
		*Stärke der Empfehlung: moderat ↑*	
Leichtere Schlaganfälle (NIHSS < 6)	AIS-Patienten mit milder Symptomatik (NIHSS 0–5) und großem Gefäßverschluss, die sich bis zu 24 h, nach dem sie zuletzt gesund gesehen wurden, vorstellen, sollten in RCTs eingeschlossen werden	Auch wenn der Nutzen unklar ist, kann bei Patienten mit einem proximalen Verschluss der vorderen Zirkulation eine MT auch bei einem NIHSS < 6 in einem Zeitfenster von 6 h durchgeführt werden	Bis zum Vorliegen besserer Evidenz sollte eine MT nur im Einzelfall nach genauer Abwägung der potenziellen Risiken und Chancen erfolgen
	*Grad der Evidenz: sehr niedrig ⊕*	*Grad der Evidenz B‑R*	
	*Stärke der Empfehlung: neutral –*	*Stärke der Empfehlung: schwach ↑?*	
Größe des Infarktes (ASPECTS)	AIS-Patienten mit einem Verschluss der vorderen Zirkulation mit großem Infarktkern (ASPECTS < 6 oder Core-Volumen > 70/100 ml) sollten in RCTs zur Evaluierung des Nutzens der MT gegenüber medizinischem Management eingeschlossen werden	Auch wenn der Nutzen unklar ist, kann bei Patienten mit einem proximalen Verschluss der vorderen Zirkulation eine MT auch bei eine ASPECTS < 6 in einem Zeitfenster von 6 h durchgeführt werden	Basierend auf der bisherigen Evidenz empfehlen wir die Erwägung einer MT bei Patienten mit einem ASPECTS von 4–6 und bei ausgewählten Patienten (frühes Zeitfenster, Alter < 70 Jahre, vorher selbstständig zu Hause lebend) auch mit einem ASPECTS von 0–3
	*Grad der Evidenz: sehr niedrig ⊕*	*Grad der Evidenz B‑R*	
	*Stärke der Empfehlung: neutral –*	*Stärke der Empfehlung: schwach ↑?*	
MT in Kombination mit i.v. tPA	AIS-Patienten mit einem LVO sollten eine kombinierte Therapie aus MT und i.v. tPA erhalten, sofern keine Kontraindikation besteht. Beide Behandlungen sollten so schnell wie möglich erfolgen und sich nicht gegenseitig verzögern	AIS-Patienten bei denen i.v. tPA infrage kommt, sollten sie erhalten, auch wenn eine MT durchgeführt werden soll	Wir empfehlen weiterhin die Gabe von i.v. tPA, auch wenn eine MT durchgeführt werden soll. Die Gabe sollte die MT auf keinen Fall verzögern Tenecteplase sollte als Alternative zur derzeit genutzten Alteplase erwogen werden, insbesondere bei Patienten mit nachgewiesenem LVO
	*Grad der Evidenz: sehr niedrig ⊕*	*Grad der Evidenz A*	
	*Stärke der Empfehlung: stark ↑↑*	*Stärke der Empfehlung: stark ↑↑*	
	Expertenmeinung: Sofern ein LVO nachgewiesen wird vor i.v. tPA-Gabe, sollte Tenecteplase bevorzugt werden	Es könnte vernünftig sein, bei MT-Patienten Tenecteplase statt Alteplase zu verwenden	
		*Grad der Evidenz B‑R*	
		*Stärke der Empfehlung: schwach ↑?*	
Distale Verschlüsse	Expertenmeinung: Bei M2-Verschlüssen besteht Konsensus unter den Experten, dass eine MT angemessen ist. Keine Aussage zu anderen distalen Verschlüssen	Bei gut ausgewählten AIS-Patienten auf Basis eines M2- oder M3-Verschlusses könnte eine MT innerhalb der ersten 6 h nach Symptombeginn von Vorteil sein	Wir empfehlen bei Patienten mit distalen Verschlüssen, die sicher per MT erreichbar sind, eine MT durchzuführen, sofern ein klinischer Vorteil zu erwarten ist. Dies gilt unabhängig vom Zeitfenster
		*Grad der Evidenz: B‑R*	
		*Stärke der Empfehlung: schwach ↑?*	
Verschlüsse der hinteren Zirkulation	Expertenmeinung: Bei Verschlüssen der A. basilaris besteht Konsensus unter den Experten, dass eine MT angemessen ist. Keine spezifische Aussage zu den Aa. posterior und vertebralis	Bei gut ausgewählten AIS-Patienten auf Basis eines Verschlusses der A. basilars, A. vertebralis oder A. posterior könnte eine MT innerhalb der ersten 6 h nach Symptombeginn von Vorteil sein	Wir empfehlen bei Verschlüssen der hinteren Zirkulation die Durchführung der MT
		*Grad der Evidenz: C-LD*	
		*Stärke der Empfehlung: schwach ↑?*	
Tandem-Verschlüsse	Der intrakranielle LVO sollte behandelt werden, hinsichtlich der Behandlungsmodalität für die extrakranielle Läsion kann keine Empfehlung gegeben werden. Diese Patienten sollten in RCTs eingeschlossen werden	Die Behandlung des/der extrakraniellen Verschlusses/Stenose während der primären Thrombektomie könnte vernünftig sein	Wir empfehlen die Durchführung der MT auch bei Tandem-Verschlüssen sowie, wenn sicher möglich, das parallele primäre Stenting der extrakraniellen Läsion
	*Grad der Evidenz: sehr niedrig ⊕*	*Grad der Evidenz B‑R*	
	*Stärke der Empfehlung: neutral –*	*Stärke der Empfehlung: schwach ↑?*	
	Expertenmeinung: 9/11 Experten sofern kein Einschluss in RCT möglich, sollte bei hochgradiger Stenose am ehesten ein Stenting erwogen werden		
Schlaganfall bei Kindern und Jugendlichen (0–17 Jahre)	Keine spezifische Empfehlung	In Abwesenheit besserer Studiendaten bleibt eine Behandlung von Kindern mit LVO durch MT umstritten [84]	Wir empfehlen die Durchführung von MT auch bei Kindern und Jugendlichen, sofern sie die Kriterien der bisherigen RCTs zur MT bei Erwachsenen erfüllen Sofern als Ursache eine Arteriopathie vermutet wird, sollte mit besonderer Vorsicht agiert werden
Schlaganfall bei sehr alten Patienten (> 90 Jahre)	Ein oberes Alterslimit für die MT ist nicht gerechtfertigt bei AIS-Patienten, die sich innerhalb von 6 h vorstellen	Der Nutzen von MT bei Patienten mit einem Alter von über 90 Jahren ist nicht klar	Wir empfehlen die MT auch bei LVO-Patienten mit einem Alter von über 90 Jahren, sofern sie vorher selbstständig waren
	*Grad der Evidenz: moderat ⊕⊕⊕*		
	*Stärke der Empfehlung: stark ↑↑*		
	ESO/ESMINT schlägt vor auch ältere LVO-Patienten (> 80 Jahre) im Zeitfenster von 6–24 h zu behandeln, sofern sie die DAWN- und DEFUSE-3-Kriterien erfüllen		
	*Grad der Evidenz: niedrig ⊕⊕*		
	*Stärke der Empfehlung: schwach ↑?*		
Vorbestehende Defizite (prämorbider mRS > 1)	Keine spezifische Empfehlung	Auch wenn der Nutzen unklar ist, könnte bei Patienten mit einem proximalen Verschluss der vorderen Zirkulation eine MT auch bei einem prämorbiden mRS > 1 in einem Zeitfenster von 6 h durchgeführt werden	Aufgrund der geringen Risiken der MT sollten vorbestehende Defizite nicht zwingend ein Ausschlussgrund für die MT sein. Wichtiger noch sollte das Ermitteln des prämorbiden mRS die Therapie nicht verzögern Wir empfehlen ein pragmatisches Kriterium, wie z. B., ob der Patient aus dem eigenen Haushalt kommt oder aus einem Pflegeheim
		*Grad der Evidenz: B‑R*	
		*Stärke der Empfehlung: schwach ↑?*	
Pre-hospital-Skalen für die Erkennung von LVOs	Keine spezifische Empfehlung. Patienten sollten, wenn möglich, in RCTs eingeschlossen werden	Es sollten effektive Pre-hospital-Skalen entwickelt werden, um Patienten mit einer hohen Wahrscheinlichkeit für einen LVO zu identifizieren, um den Transport der Patienten in ein MT-fähiges Krankenhaus zu beschleunigen	Pre-hospital-Skalen wie z. B. RACE oder Telekonsultation könnten die Triage von vermuteten LVO-Patienten verbessern. Sie sollten in Zusammenarbeit mit den Notdiensten evaluiert werden
	*Grad der Evidenz: sehr niedrig ⊕*	*Grad der Evidenz: C-EO*	
	*Stärke der Empfehlung: neutral – *	*Stärke der Empfehlung: stark ↑↑*	
Ziel der Thrombektomie	Bei Patienten mit LVO sollte versucht werden, mTICI 3 zu erreichen, sofern es möglich ist mit vertretbarer Sicherheit	Das technische Ziel der MT sollte eine Reperfusion vom Grad mTICI ≥ 2b sein	Wir empfehlen als Ziel der MT eine vollständige bzw. fast vollständige (mTICI 2c/3) Reperfusion. Wir empfehlen wiederholte Manöver auch bei bereits erreichter mTICI-2b-Reperfusion, wenn es technisch sicher möglich ist und das noch nichtperfundierte Areal funktionell wichtig ist
	*Grad der Evidenz: niedrig ⊕⊕*	*Grad der Evidenz: A*	
	*Stärke der Empfehlung: stark ↑↑*	*Stärke der Empfehlung: stark ↑↑*	

*AHA/ASA *American Heart Association/American Stroke Association, *AIS* „acute ischemic stroke“, *ASPECTS* Alberta Stroke Program CT Score, *ESO/ESMINT *European Stroke Organisation/European Society for Minimally Invasive Neurological Therapy, *mRS* modifizierte Rankin-Skala, *MT* mechanische Thrombektomie, *mTICI* „modified treatment in cerebral infarction“, *LVO* „large vessel occlusion“, *NIHSS* National Institutes of Health Stroke Scale, *RCT* randomisierte kontrollierte Studie, *tPA* „tissue plasminogen activator“

^a^Die Angaben beziehen sich auf das 2019 Update der „Guidelines for the early Managment of Patients with Acute Ischemic Stroke“ von der AHA/ASA und den 2019 veröffentlichen „ESO/ESMINT Guidelines on acute ischemic stroke.“

Die Evidenzlevel der AHA/ASA: *A* hochqualitative Evidenz von mehr als einer RCT, Metaanalyse mehrerer RCTs, eine oder mehr RCTs ergänzt durch eine hochqualitative Registerstudie. *B‑R* („randomized“) Evidenz moderater Qualität von einer oder mehr RCTs sowie von einer Metaanalyse dieser RCTs. *C‑LD* („limited data“) randomisierte oder nichtrandomisierte Studien mit Limitationen in Design oder Ausführung, Metaanalysen derartiger Studien, physiologische oder mechanistische Studien bei Menschen. *C‑EO* („expert opinion“) Konsensus von Experten basierend auf klinischer Erfahrung

## Mögliche Indikationserweiterungen für MT

### Zeitdauer zwischen Beginn der Symptome und Präsentation im Krankenhaus

In den fünf Studien, die 2015 zu einem Paradigmenwechsel in der Therapie des AIS aufgrund eines LVO der vorderen Zirkulation führten, präsentierte sich nur ein Bruchteil der Patienten (5 % von 1287 Patienten) in einem Zeitfenster von mehr als 6 h [1]. Allerdings zeigte sich bereits in der ersten Metaanalyse (HERMES) der oben genannten Studien ein statistisch signifikanter positiver Effekt auf das klinische Ergebnis (Odds Ratio [OR] 1,76; 95 %-Confidence-Intervall [CI] 1,05–2,97) auch bei Patienten, die sich mindestens 5 h nach den ersten Symptomen präsentierten [1].

Im Jahr 2018 zeigten die DAWN- und DEFUSE-3-Studien, dass bei ausgewählten Patienten die MT auch im Zeitfenster von 6–24 h zu einem deutlich besseren langfristigen neurologischen Ergebnis (definiert als „modified Rankin Scale“ [mRS] nach 90 Tagen) führt als die bisher standardmäßig genutzte medikamentöse Therapie [7, 8]. Das Besondere an diesen Studien war, dass sie als primäres Einschlusskriterium ein radiologisches Mismatch entweder zwischen dem Infarktkern und der noch „rettbaren“ Penumbra (DEFUSE‑3; [8]) oder zwischen dem Infarktkern und der klinischen Präsentation (DAWN; [7]) voraussetzten. Die sehr strengen Einschlusskriterien beider Studien führten dazu, dass aktuell nur ca. 30 % der LVO-Patienten, die sich im Zeitfenster von 6–24 h befinden, nach der aktuellen AHA/ASA-Guideline eine klare Indikation für die MT aufweisen [9]. Nichtrandomisierte Studien legen allerdings nahe, dass der Nutzen einer MT auch bei Patienten vorhanden ist, die nach den DAWN- und DEFUSE-3-Kriterien keine Kandidaten für die MT wären [9, 10]. Weiterhin konnte auch gezeigt werden, dass eine Patientenselektion auf Basis der Kollateralen zu ähnlich guten Behandlungsergebnissen führt wie die perfusionsbasierte Selektion, die in den DAWN- und der DEFUSE-3-Studien genutzt wurde [11].

Bei ausreichend „rettbarer“ Penumbra sollte die MT auch im späten Zeitfenster erwogen werden

Die aktuell laufenden Studien RESILIENT Extend (NCT02216643) und MR CLEAN LATE (ISRCTN19922220) untersuchen die Fragestellung, ob ein vereinfachtes bildgebendes Verfahren ohne CT(Computertomographie)-Perfusion oder Magnetresonanztomographie (MRT) für die Patientenselektion im Zeitfenster von mehr als 6 h genutzt werden kann. Sollten diese Studien positiv sein, würde dies zu einer deutlichen Erweiterung der Indikation für die MT führen. Weiterhin legen neuere Forschungsergebnisse nah, dass Patienten mit radiologisch nachweisbarer Penumbra von der MT selbst in einem Zeitfenster von bis zu 10 Tagen profitieren können [12]. Die MT sollte auch im sehr späten Zeitfenster bei allen LVO-Patienten erwogen werden, sofern sich ausreichende „rettbare“ Penumbra in der Perfusionsbildgebung nachweisen lässt.

### Ausgedehnte Infarktfrühzeichen

Bei fast allen RCTs waren ausgedehnte Infarktfrühzeichen, definiert als ein Alberta Stroke Program CT Score (ASPECTS) < 6, ein Ausschlusskriterium. Dennoch wurden in einigen Studien auch Patienten mit niedrigeren ASPECTS eingeschlossen. In einer 2018 veröffentlichen Metaanalyse von 7 RCTs (*n* = 1764) zeigte sich auch bei Patienten mit einem ASPECTS von 5–7 (OR 1,58; 95 %-CI 1,19–2,11) sowie mit einem ASPECTS von 0–4 (OR 2,15; 95 %-CI 1,06–4,37) ein positiver Effekt auf das neurologische Outcome. Allerdings sind insbesondere die Ergebnisse für einen ASPECTS von 0–4 aufgrund der niedrigen Stichprobe (*n* = 126) mit erheblicher Unsicherheit behaftet [13].

Damit übereinstimmend zeigte eine Metaanalyse, die auch nichtrandomisierte Studien einschloss, bei Patienten mit einem ASPECTS von 0–6 einen deutlichen Effekt der MT. So erreichten in der MT-Gruppe 30 % ein gutes neurologisches Ergebnis (definiert als mRS ≤ 2 nach 90 Tagen), während dies in der medikamentös behandelten Gruppe nur bei 3,2 % der Fall war. Subgruppenanalysen zeigten aber, dass mit steigender Infarktgröße die Quote der Patienten mit gutem neurologischem Ergebnis auch in der MT-Gruppe deutlich abnimmt. Erreichten bei den Patienten mit einem ASPECTS von 4 noch 22,1 % ein gutes neurologisches Ergebnis, so waren es in der Gruppe mit einem ASPECTS von 0–3 nur noch 13,9 % [14].

Zusammenfassend reicht die bisherige Evidenz nicht für eine abschließende Bewertung der Sicherheit und Effizienz der MT bei dieser Patientengruppe aus. Dies wurde auch in einem Konsensus-Statement der ESO, der European Society for Minimally Invasive Neurological Therapy (ESMINT) und der European Society of Neuroradiology zum Ausdruck gebracht [15]. Dies führte zur Initiierung mehrerer randomisiert-kontrollierter Studien, wie den europäischen TENSION- und LASTE-Studien (ClinicalTrials.gov NCT03094715 & NCT03811769), der amerikanischen TESLA-Studie (ClinicalTrials.gov NCT03805308) sowie der japanischen RESCUE-Japan-LIMIT (ClinicalTrials.gov; NCT03702413)-Studie.

Basierend auf der bisherigen Evidenz empfehlen wir die Erwägung einer MT bei Patienten mit einem ASPECTS von 4–6 und bei ausgewählten Patienten (frühes Zeitfenster, Alter < 70 Jahre, vorher selbstständig zu Hause lebend) auch mit einem ASPECTS von 0–3.

### Distale Verschlüsse

In den bisherigen RCTs zur Zulassung der MT (bis auf MR CLEAN und EXTEND IA) war ein wesentliches Einschlusskriterium ein LVO der vorderen Zirkulation [1, 13]. Unter einem LVO wird meistens ein Verschluss des M1-Segments der A. cerebri media sowie ein Verschluss der A. carotis interna verstanden [1]. Aufgrund der unklaren Datenlage ist ein weiter distal gelegener Verschluss der häufigste Grund dafür, keine MT durchzuführen, selbst beim Nachweis einer großen, potenziell rettbaren Penumbra [16]. Große Registerstudien (z. B. PLUMBER, STOP Stroke und INTERRSeCT Register) legen nahe, dass bei ca. 20–25 % aller AIS-Patienten ein Verschluss vorliegt, der nicht unter die oben genannte Definition des LVO fällt [17, 18]. Die Datenlage für diese sog. „medium vessel occlusions“ (MeVO, definiert als Verschluss des M2-, M3- oder M4-Segments der A. cerebri media, des A1-, A2- oder A3-Segments der A. cerebri anterior sowie des P1- und P2-Segments der A. cerebri posterior) ist trotz des häufigen Auftretens unzureichend [19].

Es wurden allerdings nur wenige (ca. 10 % der Studienpopulationen) Patienten mit einem Verschluss des M2-Segments in die bisher durchgeführten RCTs eingeschlossen, entweder aufgrund einer primär falschen Klassifikation als M1 (*n* = 71) oder da ein Einschluss laut Protokoll ausdrücklich erlaubt war (*n* = 59). In einer Metaanalyse dieser RCTs konnte bei einer Stichprobe von insgesamt 130 Patienten (67 MT-Patienten) gezeigt werden, dass die MT signifikant häufiger zu einem guten klinischen Ergebnis (definiert als mRS ≤ 2 an Tag 90) geführt hat als ein rein medikamentöses Management (absoluter Unterschied 18,5 %; *p* = 0,04) [20]. Aufgrund von Bedenken bezüglich der externen Validität dieser Metaanalyse wurden die Guidelines hinsichtlich der Behandlung des M2-Verschlusses aber weder von der ASA noch von der ESO angepasst [5, 21].

Für andere MeVOs gibt es aktuell keine Daten aus RCTs und die Ergebnisse aus retrospektiven Studien und Registern sind teilweise widersprüchlich, wobei sich in den meisten Studien kein Hinweis auf eine erhöhte Mortalität oder ein erhöhtes Risiko an Blutungen durch die MT ableiten lässt [22, 23]. Eine kürzlich veröffentliche Studie mit der bisher höchsten Anzahl an Patienten mit isoliertem P2- und P3-Verschluss (*n* = 243) lässt aber zu mindestens in der Subgruppe der schwer betroffenen (National Institute of Health Stroke Scale [NIHSS] ≥ 10) Patienten einen klinischen Effekt erwarten [24].

Nicht nur der Gefäßabschnitt, sondern auch das betroffene Hirnareal sollte betrachtet werden

Das Problem der Evaluierung von MT bei MeVOs ist auch, dass sich die von ihnen perfundierten Hirnareale hinsichtlich ihrer Größe, aber auch Funktion deutlich unterscheiden [25]. Daher sollte nicht nur der Gefäßabschnitt betrachtet werden, sondern auch welches Hirnareal betroffen ist und wie schwer die neurologischen Ausfälle sind. Insgesamt gesehen sollte die Durchführung von RCTs zu dieser Fragestellung ein ausgesprochen wichtiges Anliegen sein, da ca. 50 % der Patienten mit einem MeVO auch trotz intravenöser Thrombolyse (i.v. „tissue plasminogen activator“ [tPA]) eine bleibende relevante neurologische Behinderung entwickeln [26, 27].

Wir empfehlen bei M2-Verschlüssen die großzügige Durchführung der MT, sofern ein klinischer Vorteil für den Patienten zu erwarten ist. Bei anderen MeVOs sollte eine MT erwogen werden, sofern der Verschluss sicher erreichbar ist und das betroffene Areal von hoher funktioneller Bedeutung ist.

### Milde Schlaganfälle

Der NIHSS ist nicht nur wesentlich für die Diagnose eines Schlaganfalls, sondern auch für die weitere Therapieentscheidung [5]. Während für die i.v. tPA ein NIHSS ≥ 4 als Kriterium genutzt wird, ist bei der MT als Grenze ein NIHSS von ≥ 6 weitgehend aktzeptiert [5]. Interessanterweise waren die NIHSS-Grenzen in den bisherigen RCTs sehr variable. So war die Grenze in der MR-CLEAN-Studie 2, während die ESCAPE- und EXTEND-IA-Studien gar keine NIHSS-Grenze nutzten, aber „behindernde“ Symptome vorrausetzten [28–30]. Da aber verschiedene Studien, unter anderem die SWIFT-PRIME-, REVASCAT- und DEFUSE-3-Studie, unabhängig eine NIHSS-Grenze von 6 validierten, wurde diese in späteren Guidelines genutzt [5].

Zur Wirksamkeit der MT bei Patienten mit einem NIHSS < 6 gibt es kaum randomisiert-kontrollierte Daten. Retrospektive Daten aus dem Schweizer Schlaganfallregister (*n* = 312) sowie mehrere Multicenterstudien legen allerdings nahe, dass kein Zusatznutzen der MT gegenüber der alleinige i.v. tPA in dieser Patientengruppe besteht. Es gab aber auch keine Hinweise auf ein erhöhtes Komplikationsrisiko [31–33].

Den oben genannten Studien widersprechend weisen Daten der MINOR-STROKE-Kollaboration (*n* = 598 Patienten) darauf hin, dass milde betroffene Patienten mit einer M1-Okklusion von einer zusätzlichem MT profitieren könnten, während bei einer M2-Okklusion die alleinige i.v. tPA überlegen sein könnte [34].

Bei milden Schlaganfällen sollte die Therapieentscheidung sorgfältig abgewogen werden

Bei der Betrachtung dieser Studienergebnisse muss neben dem retrospektiven Charakter und möglichen Selektionsbias aber auch auf die inhärenten Limitationen des NIHSS hingewiesen werden. Der NIHSS bildet nicht das Ausmaß der individuellen Behinderung durch die neurologischen Defizite ab. So würde eine Dysarthrie im NIHSS nur einem Punktwert von 1 entsprechen, aber im Falle einer Persistenz bei Lehrern und Anwälten zu gravierenden Langzeitfolgen führen. Ähnliches gilt für eine Schwäche der Hand bei einem Musiker oder Feinmechaniker. Basierend auf diesen Gedankengängen kann bei milden Schlaganfällen keine allgemeine Empfehlung ausgesprochen werden und die Therapieentscheidung sollte nach sorgfältiger Abwägung des möglichen Nutzens und der Risiken getroffen werden. Da Patienten mit einem NIHSS < 6 meistens noch kommunizieren können, sollte diese Entscheidung idealerweise in Absprache mit dem Patienten getroffen werden.

### Verschluss der A. basilaris

Verschiedene Metaanalysen retrospektiver Studien legen nahe, dass die MT auch bei Verschlüssen der A. basilaris gegenüber einer rein medikamentösen Therapie zu einem verbesserten langfristigen neurologischen Ergebnis führt, ohne dabei mit erhöhten Komplikationsraten einherzugehen [35, 36]. Zu diesem Ergebnis kam auch eine jüngst veröffentliche Registerstudie mit 829 Patienten. In dieser Studie erhöhte die MT die Wahrscheinlichkeit für ein gutes klinisches Ergebnis (mRS ≤ 3) deutlich [37]. Dem gegenüber stehen Ergebnisse aus zwei RCTs (BEST und BASICS), die keinen signifikanten positiven Effekt der MT auf das klinische Ergebnis bei Verschlüssen der A. basilaris zeigen konnten [38, 39]. Es muss allerdings beachtet werden, dass in beiden Studien initial heute nicht mehr genutzte Katheter verwendet wurden und das es aufgrund zwischenzeitlich neu verfügbarer Daten zu hohen Cross-over-Raten (von konservativ zu MT) und verminderter Rekrutierung (Die BEST-Studie wurde früher als geplant beendet) kam [38, 39]. Daher kann aus diesen Studien keine definitive Empfehlung abgeleitet werden, vor allem bei schwer betroffenen Patienten mit kleinem Infarktkern.

Leider ist auch für die Zukunft nicht zu erwarten, dass diese Fragestellung durch eine RCT geklärt wird, da aufgrund des starken Effekts der MT bei anderen Verschlüssen starker Widerstand gegen eine Randomisierung zu erwarten ist. Aus diesem Grund und auf Basis unserer klinischen Erfahrung empfehlen wir im Kontrast zu den zwei RCTs die MT für Verschlüsse des vertebrobasilaren Systems, vor allem bei schwer betroffenen Patienten mit kleinem Infarktkern.

### Tandem-Verschlüsse

Unter einem Tandem-Verschluss versteht man die Kombination aus einem intrakraniellen Verschluss (z. B. des M1-Segments) und einer/eines extrakraniellen Stenose/Verschlusses (z. B. der A. carotis interna [ACI]) im gleichen Stromgebiet. Diese Verschlüsse sind technisch schwerer zu behandeln und das klinische Ergebnis ist meistens schlechter als bei isolierten Verschlüssen [40].

In der HERMES-Analyse wurden 122 Patienten (von 1254) mit einem Tandem-Verschluss eingeschlossen, es wurde aber keine Subgruppenanalyse für diese Patientengruppe durchgeführt [1]. Weiterhin war die Behandlung des extrakraniellen Verschlusses nicht standardisiert und variierte (keine Rekanalisation, Angioplastie oder Stenting). Eine große retrospektive Multicenterstudie (*n* = 482 Patienten), durchgeführt von der „Thrombectomy-in-Tandem-Lesions“(TITAN)-Gruppe, legt nahe, dass primäres Stenting der extrakraniellen Läsion die beste Chance auf eine erfolgreiche Rekanalisation beider Verschlüsse bietet (mTICI [„modified treatment in cerebral infarction“] ≥ 2b-Rate von 79,4 % gegenüber nur 60,2 % bei reiner MT; [41]). Es konnte zwar kein signifikanter Effekt auf das klinische Ergebnis gezeigt werden, das Komplikationsrisiko war aber ebenfalls nicht erhöht [42]. Aufgrund des Mangels an hochqualitativen Daten hinsichtlich der optimalen Behandlungsstrategie für Tandem-Verschlüsse planen die TITAN-Kollaborateure, eine RCT zu starten [43].

Basierend auf diesen vorläufigen Daten sowie vielversprechenden neuen Techniken für die Behandlung von Tandem-Verschlüssen [44] empfehlen wir die Durchführung einer MT bei Tandem-Verschlüssen mit intrakraniellem LVO sowie bei ausgewählten Patienten das primäre Stenting der extrakraniellen Läsion.

### Pädiatrische Schlaganfälle (0–17 Jahre)

Auch wenn der AIS mit einer Inzidenz von nur ca. 2 bis 8 pro 100.000 Kindern pro Jahr ein seltenes Ereignis ist, ist er dennoch ein wesentlicher Grund für langfristige Behinderungen und den Verlust an Lebensjahren [45]. Bis vor kurzem war die Evidenz für die MT in pädiatrischen Populationen (0–17 Jahre) auf kleine Fallserien beschränkt. Dies hat sich 2020 durch die Publikation zweier Arbeiten verändert, einer Metaanalyse basierend auf individuellen Patientendaten (*n* = 110) sowie der retrospektiven Multicenterstudie Save ChildS (*n* = 73). Beide Studien zeigen übereinstimmend ermutigende Daten sowohl hinsichtlich des angiographischen Erfolges (jeweils mTICI ≥ 2b-Raten von über 80 %) als auch hinsichtlich des klinischen Ergebnisses bei einem vertretbaren Sicherheitsprofil [46, 47]. Eine sekundäre Analyse der Save ChildS Daten zeigte weiterhin, dass die klinisch neurologischen Ergebnisse generell gut sind unabhängig von Ätiologie, Alter und damit verbundener MT-Technik [48]. Weiterhin konnte gezeigt werden, dass analog zu Erwachsenen die MT auch bei pädiatrischen Patienten im erweiterten Zeitfenster (6–24 h) zu einem guten neurologischen Ergebnis führt, sofern ein relevantes Mismatch zwischen Infarktkern und Penumbra besteht [49].

Auch wenn die genannten Studien nicht an die Qualität von RCTs heranreichen, werden in absehbarer Zeit keine Daten höherer Qualität verfügbar sein. Unterstrichen wird diese Annahme durch das Scheitern der Thrombolysis-in-Pediatric-Stroke(TIPS)-Studie, die aufgrund fehlender Patientenrekrutierung nach über einem Jahr Studiendauer und 14 aktivierten Zentren abgebrochen wurde. Ähnliches wäre bei einer RCT zur MT bei pädiatrischen AIS-Patienten zu erwarten, da in Anbetracht des sehr großen Effektes bei erwachsenen Patienten kaum Ärzte und auch Eltern bereit wären, Kinder zu randomisieren.

Zusammenfassend empfehlen wir insbesondere aufgrund des günstig erscheinenden Risikoprofils die MT bei pädiatrischen Patienten mit LVO, selbst im verlängerten Zeitfenster von 6–24 h. Vorsicht ist aber bei einem begründeten Verdacht auf eine Arteriopathie angebracht.

### MT bei sehr alten Patienten (> 90 Jahre)

Die aktuelle AHA/ASA-Guideline gibt keine genaue Altersgrenze nach oben für die Durchführung der MT vor, allerdings fehlen bei Patienten mit einem Alter von über 90 Jahren randomisierte Daten und so kann aktuell keine abschließende Aussage zur Wirksamkeit der MT bei Patienten in diesem Alter getroffen werden [5]. Nichtrandomisierte Studien legen allerdings einen positiven Effekt der MT auch in dieser Patientengruppe nahe [50].

Der Großteil der Ärzte entscheidet sich auch bei Patienten > 90 Jahren für eine MT

So zeigte sich in einer Analyse von 124 über 90 Jahre alten Patienten aus dem ETIS-Register, dass die Chancen auf ein gutes neurologisches Ergebnis (definiert als mRS ≤ 3 nach 90 Tagen) signifikant höher waren, sofern eine erfolgreiche Rekanalisation erreicht wurde [51]. Weiterhin konnte in bisherigen Analysen kein erhöhtes Risiko für Komplikationen in dieser Altersgruppe nachgewiesen werden [1, 51]. Passend dazu zeigte eine Umfrage unter 607 Neuroradiologen und Neurologen, dass der Großteil (74 %) sich auch bei Patienten über 90 Jahren für eine MT entscheidet, sofern eine klare Indikation (Level 1A) vorliegt [52].

Zusammenfassend sollte das Alter aus unserer Sicht kein Ausschlussgrund für die MT sein, insbesondere bei Patienten, die vor dem Schlaganfall eine gute Lebensqualität hatten.

### Patienten mit vorbestehenden Behinderungen (mRS > 1)

Aufgrund der Einschlusskriterien der bisher durchgeführten RCTs gibt es bisher kaum Daten zum Effekt der MT bei Patienten mit einem mRS > 1. So war nur in der MR-CLEAN-Studie ein mRS > 1 kein Ausschlusskriterium für die MT und es wurden 21 Patienten mit vorbestehender Behinderung eingeschlossen [28]. Da diese Patienten allerdings nicht gesondert analysiert wurden, lässt sich aktuell keine Aussage über diese Patientengruppe treffen. Dies ist insbesondere problematisch, da Patienten mit einem mRS > 1 ca. 30 % aller Patienten mit einem LVO ausmachen [53]. Kleinere retrospektive Studien legen allerdings nahe, dass bei ungefähr einem Drittel der prämorbide Status erhalten werden kann, was dem Ziel der MT entspricht [54]. Dies konnte jüngst von Salwi et al. in einer größeren Kohorte (*n* = 259) bestätigt werden. Insgesamt konnten sie dabei keine ausreichenden Belege dafür finden, dass sich das klinische Ergebnis von Patienten mit prämorbider moderater Behinderung (mRS 2–3) von Patienten mit leichter bis keine Behinderung (mRS 0–1) unterscheidet [55].

Wir empfehlen daher, dass das Erheben des prämorbiden Status für die Therapieentscheidung eine untergeordnete Rolle spielen sollte und in keinem Fall zu einer Verzögerung der MT führen darf, da der Effekt der MT durch intrahospitale Zeitverzögerungen dramatisch abnimmt [56]. Hier wäre ein pragmatischer Ansatz als Kriterium den Lebensort des Patienten zu nutzen, z. B. kommt aus eigenem Haushalt vs. Pflegeheim.

## Neue Entwicklung in Behandlung und Management von LVO-Patienten

### Intravenöse Thrombolyse bei MT-Patienten

Die Zulassung der i.v. tPA erfolgte 1995, nachdem ein deutlich verbessertes klinisches Ergebnis durch i.v. tPA bei AIS-Patienten im Zeitfenster von 3 h nach Symptombeginn gezeigt werden konnten. Durch spätere Studien konnte dieses Zeitfenster auf 4,5 h erweitert werden und auch bei ausgewählten AIS-Patienten bei unbekanntem Zeitfenster ist die i.v. tPA bei passender Bildgebung mittlerweile indiziert [57, 58]. Durch diesen historischen Zusammenhang wurde die MT bisher in RCTs nur als Zusatz zur i.v. tPA evaluiert [1]. Allerdings weisen neuere nichtrandomisierte Daten darauf hin, dass die i.v. tPA bei MT-fähigen Patienten keinen positiven Zusatznutzen haben könnte bzw. sogar das Blutungsrisiko erhöhen könnte [59]. Es gibt allerdings auch Daten, die auf einen möglichen positiven Effekt der i.v. TPA in diesem Patientenkollektiv hinweisen [60]. Erschwert wird die Bewertung der Effektivität der i.v. tPA durch den Umstand, dass die i.v. tPA je nach Thrombuslokalisation und Thrombuszusammensetzung unterschiedlich effektiv ist [61]. Interessanterweise kann auch nicht von einer Verbesserung der MT-Ergebnisse durch Weglassen der i.v. tPA auf ein besseres klinisches Ergebnis geschlossen werden. Daten aus zwei multizentrischen Kohorten weisen sogar daraufhin, dass das klinische Ergebnis bei Patienten nach Kombinationstherapie besser ist, obwohl das finale MT-Ergebnis schlechter war [62, 63]. Diese Daten sind wichtig, da das primäre Ziel einer Therapie immer das klinische Ergebnis sein muss und kein Surrogatparameter, wie das Reperfusionsergebnis.

Die beste derzeit verfügbare Evidenz stammt aus drei kürzlich veröffentlichen RCTs. Diese haben untersucht, ob die alleinige MT der Kombinationstherapie hinsichtlich des klinischen Ergebnisses (mRS nach 90 Tagen) nicht unterlegen ist. Während dies die in Japan durchgeführte SKIP-Studie nicht zeigen konnte [64], war die alleinige MT der Kombinationstherapie in den zwei chinesischen Studien entsprechend der vordefinierten Kriterien (DIRECT-MT [65], DEVT [66]) nicht unterlegen. Aufgrund der widersprüchlichen Ergebnissen, deutlich verlängerten Zeitspannen (z. B. Dauer von 59 min zwischen Patientenankunft und Bolusgabe) in den chinesischen Studien sowie auch abweichenden Schlaganfallätiologien in asiatischen Populationen haben die amerikanischen und europäischen Fachgesellschaften ihre Guidelines nicht angepasst [67]. Aktuell werden in Europa zwei RCTs mit dieser Fragestellung durchgeführt: SWIFT DIRECT (NCT03192332) und MR CLEAN-NOIV (ISRCTN80619088). Es gilt allerdings, dass die oben genannten Überlegungen nur für Patienten gelten, die sich direkt im MT-fähigen Krankenhaus vorstellen.

Die iv. tPA-Gabe sollte auf keinen Fall die MT verzögern

Ein weiterer wesentlicher Unsicherheitsfaktor ist, ob die Studienergebnisse auch auf die bisher nicht standardmäßig verwendete Tenecteplase übertragbar sind, da diese laut einer kürzlich veröffentlichen RCT der Alteplase hinsichtlich früher Rekanalisation sowie klinischem Ergebnis überlegen ist [68]. Weiterhin ist Tenecteplase auch aufgrund der Möglichkeit zur Einmalgabe als Bolus Alteplase überlegen [68].

Bis zum Ergebnis dieser Studien empfehlen wir weiterhin in Übereinstimmung mit aktuellen Guidelines die kombinierte Therapie bei geeigneten Patienten, ggf. sollte aber bei bestehender Zulassung auf Tenecteplase gewechselt werden. Die iv. tPA-Gabe sollte aber auf keinen Fall die MT verzögern.

### Neue Triage-Konzepte

Bei AIS-Patienten ist die Zeit zwischen Symptom- und Behandlungsbeginn von herausragender Bedeutung, allerdings besteht je nach Ätiologie des AIS (MT geeignet vs. nur Lysetherapie geeignet) ein Zielkonflikt im Hinblick auf die Triage vor der Krankenhauseinlieferung [69]. Während ein AIS-Patient mit einem Verschluss, der für die MT nicht zugänglich ist, in das nächste lysefähige Krankenhaus transferiert werden sollte, ist für einen Patienten mit LVO ein Transfer in das nächstgelegene MT-fähige Krankenhaus von Vorteil. Dies gilt auch, wenn die primäre Transportzeit bis zu 30 min länger ist [70].

Aus diesem Grund haben verschiedene Autorengruppen Skalen für die Detektion von LVOs entwickelt. Wesentlich hier zu nennen sind die Rapid-Arterial-Occlusion-Evaluation(RACE)-Skala, der Los Angeles Motor Score (LAMS), die Gaze-Face-Arm-Speech-Time(G-FAST)-Skala und die Field-Assessment-Stroke-Triage-for-Emergency-Department(FAST-ED)-Skala [71]. Zwei kürzlich veröffentliche prospektive, multizentrische Studien mit jeweils über 1000 Patienten konnten für jede dieser Skalen eine akzeptable bis gute Genauigkeit zeigen, wobei in beiden Studien die RACE-Skala die höchste Genauigkeit hatte [71, 72]. Insgesamt zeigt sich aber weiterhin, dass der von Ärzten erhobene NIHSS die höchste Genauigkeit hat [71]. Eine Kombination beider Techniken durch Telekonsultation im Krankenwagen wurde daher in Stockholm erprobt und hat dort zu deutlich verkürzten Transferzeiten geführt sowohl für die i.v. tPA als auch für die MT [73]. Eine Gruppe des Universitätsspitals Basel evaluiert derzeit einen ähnlichen Ansatz in der Telestroke-2-Studie (NCT04578002). Eine weitere technische Möglichkeit zur optimalen Triage von AIS-Patienten außerhalb des Krankenhauses ist die Nutzung von „mobile stroke units“. Für diese konnte in einer Metaanalyse bereits eine deutlich reduzierte Zeit bis zum Behandlungsbeginn gezeigt werden [74]. Allerdings bestehen aufgrund der hohen Kosten noch erhebliche Widerstände gegen eine breite Einführung.

Das OneStop-Management führt zu erheblichen Zeitersparnissen

Zur Minimierung der Zeit von der Krankenhaustür bis zum Beginn der MT wurden ebenfalls neue Triage- und Workflow-Konzepte entwickelt. Ein wesentlicher Ansatz dafür ist das OneStop-Management, bei dem der Patient direkt in die Angiographieeinheit verbracht wird und dort die primäre Bildgebung stattfindet [75]. Dadurch wird ein „stop“ im Multidetector-CT(MDCT)- bzw. MRT-Raum eingespart. Untersuchungen zeigen, dass in vielen Krankenhäusern erhebliche Distanzen zwischen dem CT/MRT- und dem Angiographieraum besteht [76]. Aber selbst in Krankenhäusern, bei denen Angiographie- und MDCT-Raum nebeneinander sind, führt das OneStop-Management zu erheblichen Zeitersparnissen (Median 25 min [Interquartilsabstand (IQR) 19–30] vs. Median 60 min [IQR 48–68]; *p* < 0,001; [77]).

Zusammenfassend empfehlen wir die Einführung einfach durchzuführender Pre-hospital-Scores wie z. B. der RACE-Skala, um eine verbesserte Triage von AIS-Patienten zu erreichen.

### Technisches Ziel der MT

Allgemein wird eine MT als „erfolgreich“ definiert, sofern am Ende der Prozedur ein mTICI von mehr als 2b erreicht wurde, dies entspricht einer Reperfusion von mehr als 50 % des vorher nichtperfundierten Areals [78]. Aufgrund technischer Fortschritte und der Erkenntnis, dass die Reperfusion einer der wichtigsten Einflussfaktoren für das langfristige klinische Ergebnis ist, wurde von verschiedenen Autorengruppen vorgeschlagen, dass ein mTICI-Score von 2c oder 3 (Perfusion von ≥ 90 % des vorher nichtperfundierten Areals) als neue Definition genutzt werden sollte [79, 80]. Es konnte in mehreren retrospektiven Studien gezeigt werden, dass eine sekundäre Verbesserung des mTICI 2b auf 2c/3 zu einer Verbesserung des klinischen Ergebnisses führt [81]. Dies gilt auch für den Fall, dass sich die Prozedur dadurch verlängert [82]. Allerdings muss hier auf einen möglichen Selektionsbias hingewiesen werden, da aus den Arbeiten nicht klar hervorgeht, warum bei einem Teil der Patienten eine Verbesserung des MT-Ergebnisses angestrebt wurde und bei den anderen Patienten nicht. Insgesamt wären zu dieser Fragestellung RCTs wünschenswert, um abschließend zu klären, ob eine Verbesserung des MT-Ergebnisses tatsächlich zu klinisch besseren Ergebnissen führt oder ob sie mit einem erhöhten Komplikationsrisiko einhergeht.

Zusammenfassend empfehlen wir aktuell die Verbesserung des angiographischen Ergebnisses bei ausgewählten Patienten, hierbei sollten in Übereinstimmung mit einem kürzlich erschienen Expertenstatement primär folgende Fragestellungen eine Rolle spielen:

- Ist das nichtperfundierte Areal bereits infarziert?
- Welche funktionelle Bedeutung hat es?
- Wie hoch sind die möglichen Risiken bei einem weiteren MT-Manöver [83]?

## Fazit für die Praxis

- Zusammenfassend empfehlen wir die mechanische Thrombektomie (MT) unabhängig vom Zeitfenster zu erwägen, auch wenn die DAWN- und DEFUSE-3-Kriterien nicht erfüllt sind, sofern noch ausreichend Penumbra nachweisbar ist.
- Zusätzlich sollten auch Patienten mit einem Verschluss der A. basilaris oder einem Tandem-Verschluss nicht von der MT ausgeschlossen werden. Dies gilt unserer Einschätzung nach auch für sehr alte (> 90 Jahre) und junge (0–17 Jahre) Patienten.
- Bei Patienten mit vorbestehender Behinderung oder bereits sehr ausgedehnten Infarktzeichen sollte die MT weiterhin eine Einzelfallentscheidung darstellen.
- Für die Zukunft gehen wir davon aus, dass sich die Indikation für eine MT aufgrund technischer Verbesserungen deutlich erweitern wird, vor allem auf distal gelegene, kleine Verschlüsse.
